# Kidney Protection with the Radical Scavenger α_1_-Microglobulin (A1M) during Peptide Receptor Radionuclide and Radioligand Therapy

**DOI:** 10.3390/antiox10081271

**Published:** 2021-08-10

**Authors:** Amanda Kristiansson, Anders Örbom, Oskar Vilhelmsson Timmermand, Jonas Ahlstedt, Sven-Erik Strand, Bo Åkerström

**Affiliations:** 1 Department of Clinical Sciences Lund, Oncology, Lund University, 221 00 Lund, Sweden; anders.orbom@med.lu.se (A.Ö.); oskar.vilhelmsson_timmermand@med.lu.se (O.V.T.); sven-erik.strand@med.lu.se (S.-E.S.); 2 Department of Clinical Sciences Lund, CIPA, Lund University, 221 84 Lund, Sweden; jonas.ahlstedt@med.lu.se; 3 Department of Clinical Sciences Lund, Medical Radiation Physics, Lund University, 221 00 Lund, Sweden; 4 Department of Clinical Sciences Lund, Section for Infection Medicine, Lund University, 221 84 Lund, Sweden; bo.akerstrom@med.lu.se

**Keywords:** A1M, radical scavenger, radionuclide therapy, kidney protection, PSMA, dosimetry

## Abstract

α_1_-Microglobulin (A1M) is an antioxidant found in all vertebrates, including humans. It has enzymatic reductase activity and can scavenge radicals and bind free heme groups. Infused recombinant A1M accumulates in the kidneys and has therefore been successful in protecting kidney injuries in different animal models. In this review, we focus on A1M as a radioprotector of the kidneys during peptide receptor radionuclide/radioligand therapy (PRRT/RLT). Patients with, e.g., neuroendocrine tumors or castration resistant prostate cancer can be treated by administration of radiolabeled small molecules which target and therefore enable the irradiation and killing of cancer cells through specific receptor interaction. The treatment is not curative, and kidney toxicity has been reported as a side effect since the small, radiolabeled substances are retained and excreted through the kidneys. In recent studies, A1M was shown to have radioprotective effects on cell cultures as well as having a similar biodistribution as the somatostatin analogue peptide ^177^Lu-DOTATATE after intravenous infusion in mice. Therefore, several animal studies were conducted to investigate the in vivo radioprotective potential of A1M towards kidneys. The results of these studies demonstrated that A1M co-infusion yielded protection against kidney toxicity and improved overall survival in mouse models. Moreover, two different mouse studies reported that A1M did not interfere with tumor treatment itself. Here, we give an overview of radionuclide therapy, the A1M physiology and the results from the radioprotector studies of the protein.

## 1. Introduction

Acute kidney injury (AKI) can occur in conjunction with a wide array of diseases and medical conditions inducing various forms of stress to this organ [1]. Iatrogenic AKI also occurs as a result of medical interventions and treatments such as major surgery or radiation treatment [2,3]. The pathogenic factors involved in the development of AKI are incompletely mapped and understood but are probably as diverse as the number of conditions that are associated with AKI. However, in most forms of AKI, if not all, oxidative stress is involved [4].

Oxidative stress is a general term covering physiological conditions with a high production of oxidative compounds and/or impaired antioxidation defenses in biological systems [5]. The mediators of oxidative stress include free radicals and reactive oxygen species (ROS). These are produced in cells and tissues in normal biological processes, such as metabolism, oxygen transport, immune response, etc., and at increased rates during inflammatory disease conditions. The free radicals and ROS are highly reactive due to the presence of unpaired electrons. During oxidative stress, a net excess of free radicals and ROS react with proteins, DNA, membranes, etc., in an uncontrolled manner, leading to unwanted modifications of the target molecules and loss of function.

In health, ROS and free radicals are counteracted by an arsenal of antioxidants, including enzymes such as superoxide dismutase, glutathione peroxidase and catalase [6,7,8]. One of the most recently described components of the endogenous antioxidation defense system, α_1_-microglobulin (A1M), is a small protein found both intra- and extracellularly in all tissues of vertebrates [9]. A1M acts both by reduction and scavenging of ROS and radicals and by reduction of oxidized macromolecules. A1M is synthesized and secreted by all cells of the body but at a higher rate in the liver. A rapid flow of the protein through blood and extravascular compartments ensures clearing of biological fluids from free radicals and ROS and repair of oxidative lesions.

In this review, the impact of radiation-based cancer therapy modalities, peptide receptor radionuclide therapy (PRRT) and radioligand therapy (RLT), on kidney function will be discussed. In both of these, infused radiolabeled small molecules target metastatic tumors through specific receptor interaction, resulting in killing of the tumor cells by irradiation damage [10]. The small-sized radiolabeled ligands, however, also accumulate in the kidneys due to glomerular filtration and tubular reabsorption, and therefore, therapeutic absorbed doses must be kept low to avoid kidney damage [11]. A1M has been shown to protect bystander cells and tissues against radiation damage, and infused A1M is predominantly distributed to the kidneys. Therefore, the protein has been suggested as a potential kidney radioprotector during PRRT and RLT. Here, the use of A1M as a renal protector will be discussed in the light of recent promising results in in vivo mouse models.

## 2. Peptide Receptor Radionuclide and Radioligand Therapy

### 2.1. Oxidative Stress

**Figure 1:**
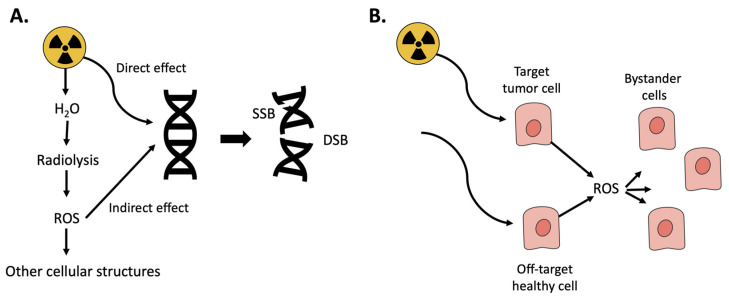
Oxidative stress resulting from ionizing radiation. (**A**) Radiation can have either direct effect resulting in DNA-damage, single or double-strand breaks (SSB/DSB) or react with water in a process called radiolysis resulting in ROS. (**B**) Radiation is used to kill tumor cells; however, it can also damage healthy cells either by directly hitting them or by the resulting ROS.

When a biological tissue is irradiated with ionizing radiation, ionizations and excitations occur resulting in generation of ROS and oxidative stress. For low LET (linear energy transfer) irradiation (photons and electrons), most of the biological consequences are caused by so called indirect effects, meaning that radicals and ROS produced by water molecules by radiolysis can diffuse to the DNA and cause damage in the form of single-strand breaks and double-strand breaks (DSB). For high LET irradiation (alpha), most of the damage is caused by direct effects, hits, on the DNA. DSB are the most severe forms of DNA-damage [12]. If reparation processes by the cellular DNA repair systems fail, DSB result in genomic instability, cell death, or cellular senescence [13]. Radiation therapy, e.g., PRRT and RLT, utilizes this effect to target and kill cancer cells. However, even if treatments today are precise, off-target effects cannot be avoided completely and healthy cells in the proximity are also damaged [14]. Moreover, radiation-induced ROS can also damage nearby cells, bystander cells, and trigger signaling pathways that initiate, e.g., inflammation (Figure 1) [15]. In PRRT and RLT, the radioactive peptides/ligands are retained and cleared through the kidneys [16,17], and the resulting radiation-induced acute cell death, inflammation and cellular senescence can lead to chronic inflammation with resulting renal fibrosis [18]. This may progress kidney pathology towards chronic kidney disease (CKD) [19]. Of importance in this paper, oxidative stress is thus involved in the generation of renal damage during PRRT and RLT.

### 2.2. Dosimetry

The energy imparted by ionizing radiation to a tissue, the absorbed dose, is measured in Gray (Gy) and defined as Joule deposited per kilogram of material (1 J/kg = 1 Gy). A more sophisticated measure, also with the unit in Gray, is the biologically effective dose (BED). BED is based on biological knowledge of cell survival after irradiation and takes into account the total absorbed dose, the absorbed dose-per-fraction of treatment, and the total time of irradiation [20]. A comprehensive summary of nuclear medicine dosimetry is given in ICRU 67 [21]. Absorbed dose estimations are usually performed using the MIRD scheme [22]. The operator will measure the amount of radioactivity in the tissue of interest at different points in time, either through tissue sampling or imaging and then calculate the number of decays that have occurred over time in a tissue. Thereafter, pre-calculated conversion factors, so called S-values, are used to convert the number of decays to absorbed dose in both the source tissue and nearby tissues that have been irradiated [22]. Alternative clinical methods to calculate the absorbed dose exist, such as measuring distributed injected radioactivity of a patient using imaging techniques, i.e., computed tomography (CT) and single-photon emission CT (SPECT) and using Monte Carlo simulation or pre-calculated dose kernels to calculate a 3D map of absorbed dose in tissues [23,24,25].

Most clinics use an approach of administering a standard activity per treatment without individual patient dosimetry. Such dosimetry, however, can be done with two [24], or only one [26], instance of SPECT/CT imaging of the patient post treatment, to estimate, i.e., the kidney absorbed dose. Use of dosimetry for radionuclide therapy in Europe has been reported by Sjögreen et al. [27].

### 2.3. Somatostatin Receptor Targeting

There are several subtypes of Somatostatin receptors (SSTRs) in humans. SSTR targeted radionuclide therapy is a treatment modality used against neuroendocrine tumors (NETs) and gastroenteropancreatic neuroendocrine neoplasms (NENs). Radiolabeled somatostatin analogues (SSAs) were initially developed for the purpose of imaging SSTR type 2 (SSTR-2) expressions in gastroenteropancreatic (GEP)-NETs as these generally express SSTR-2 correlated to tumor grade [28,29]. Targeting NETs with therapeutic radiolabeled SSAs has been done for almost three decades [30]. Unlabeled SSAs such as octreotide and lanreotide that bind to the SSTR are used to reduce clinical symptoms, i.e., carcinoid syndrome, of NETs as they inhibit the secretion of gastrointestinal hormones and potentially reduce tumor growth [31]. There are several different radiopharmaceuticals targeting the SSTR-2, based on octreotide and other SSAs modified with bifunctional chelates for radiolabeling. The NETTER-1 study, a phase 3 clinical study on [^177^Lu]Lu-DOTATATE (Lutathera), showed that ^177^Lu labeled SSAs can provide a higher progression free survival rate at 20 months compared to octreotide long-acting repeatable (LAR), a common first line therapy for NETs. The median progression free survival was not reached for the Lutathera treated arm during the studied 20 months as compared to 8.4 months for LAR [32].

### 2.4. Kidney Dosimetry in ^177^Lu-Octreotate Therapy

There are established maximum tolerable dose (MTD) absorbed dose thresholds from the field of external radiotherapy. Here it has been found that 23 Gy to both kidneys can cause chronic kidney disease in 5% of irradiated patients, five years post irradiation, while 28 Gy will cause it in 50% [33]. However, in the Quantitative Analyses of Normal Tissue Effects in the Clinic (QUANTEC) study, the 50% level was reached already when both kidneys received an absorbed dose higher than 18 Gy. In external radiotherapy, the fractionation of absorbed dose will affect the MTD, and it is estimated that an acute absorbed dose of only 4 Gy may lead to renal injury [18].

Applying MTDs from external radiotherapy onto PRRT and RLT is difficult, in part because of the different time scale of which the irradiation is conducted (seconds or minutes vs. hours or days). However, if therapies were based on a MTD to the kidney and individual dosimetry instead of a prescription of standard activity, the absorbed dose to the tumor in ^177^Lu-octreotate therapy could potentially be raised by a factor of 1.47 [24].

In a 2018 review, the median kidney absorbed dose per injected activity found in published literature was 3.3 Gy/GBq for ^90^Y-DOTATOC and 0.8 Gy/GBq for ^177^Lu-DOTATATE. The same review also found a dose–response relationship for kidney impairment, especially using BED and that, even though diverse criteria were used in different studies, ^177^Lu-labeled therapy agents had consistently lower kidney toxicity than ^90^Y-labeled ones [34].

In a study using ^177^Lu-DOTATATE, half of the patients did not reach absorbed dose 23 Gy to the kidney after 4 fractions of 7.4 GBq [24]. In another study with the same therapeutic agent, the mean absorbed dose to kidneys was 23.8 ± 6.5 Gy, but no correlation was found between absorbed dose and a decrease in the tubular extraction rate determined by ^99m^Tc-MAG3 renal scintigraphy [35]. By analyzing creatinine clearance loss in patients treated with ^90^Y-DOTATOC, it was determined that for patients without risk factors such as hypertension, diabetes or renal morphological abnormalities, a BED of 40 Gy to the kidneys could be reached before any renal toxicity. For patients with these risk factors, the BED threshold was 28 Gy. The fewer the fractions that the absorbed dose had been delivered over, the more creatinine clearance loss occurred [36].

### 2.5. Targeting Prostate-Specific Membrane Antigen (PSMA)

Prostate-specific membrane antigen (PSMA) is a 100-kDa type II transmembrane glycoprotein that is overexpressed in nearly all prostate cancers (PCa), and in particularly poorly differentiated and metastatic lesions, with only 5–10% of primary PCa lesions shown to be PSMA-negative [37]. Its expression increases in higher-grade, metastatic, and androgen-insensitive tumors, whereas expression is largely absent in benign or hyperplastic prostate tissue [38]. The name PSMA is a misnomer, as low PSMA expression also occurs in proximal small bowel, kidneys, and salivary and lacrimal glands. In the salivary glands, staining of PSMA protein is found in focal hot spots of acinar cells preferably along their luminal border but not homogeneously dispersed within the parenchyma. In the kidneys, the PSMA protein is found in a selected subset of tubular cells with a predominant luminal staining pattern [39]. Expression in malignancies is not limited to PCa; PSMA expression is also found in other types of malignancies, e.g., breast and colon carcinoma.

The introduction of a radiolabeled ligand to PSMA, ^68^Ga-PSMA-11, represented the first clinical breakthrough in PET imaging of PSMA expression [40]. It is currently the most widely used imaging PSMA-ligand. Several other radiolabeled small-molecule inhibitors of PSMA have been proposed [41].

Therapy in patients with PSMA targeting radiopharmaceuticals has been performed implementing the β-emitter ^177^Lu or the α-emitter ^225^Ac. Promising results after therapy with ^177^Lu-PSMA-617 in patients with metastatic castration-resistant PCa (mCRPC) have been reported [42,43]. Here, ^177^Lu-PSMA-617 therapy showed low hematotoxicity and no severe nephrotoxicity with only mild to moderate xerostomia (dry mouth due to saliva loss) in 8% of the patients with an administered activity of 6 GBq. Targeted therapy with ^225^Ac-PSMA has shown to be superior to ^177^Lu-PSMA both in terms of therapeutic efficacy and adverse effects [39].

### 2.6. PSMA Radioligand Therapy Dosimetry

Few radionuclide treatments up to date for PCa have involved the use of dosimetry either to plan treatment or to retrospectively ascertain the absorbed dose during treatment. Thus, few reports exist for correlation between absorbed dose and biological effect. In PSMA targeted therapy, standard activity and peptide amount are usually administered, although the tumor burden varies considerably (from milliliters to several liters). Optimization of therapy with mathematical models has been reported by Begum et al. [44], and it was shown that the BEDs to normal tumor tissue could vary considerably in patients treated with ^177^Lu-PSMA.

Literature data for kidney dosimetry can be summarized as for beta emitters ^177^Lu-PSMA-617 0.01–0.11 mGy/MBq [45,46,47,48,49,50,51,52], ^177^Lu-PSMA-I&T 0.72 mGy/MBq [53], and ^177^Lu-DOAT^ZOL^ 0.49 mGy/MBq [54] and for alpha emitters ^225^Ac-PSMA-617 0.7 mSv/MBq [39] and ^213^Bi-PSMA-617 8.1 mSv/MBq [55].

Dosimetry of kidney and tumors was investigated in patients with only one kidney [56]. The single functioning kidney presented high activity of ^177^Lu-PSMA-617. The patients were given between 2 to 6 cycles of therapy with between 15–34 GBq. The mean injected activity per cycle was 6.39 ± 1.05 GBq. The kidney absorbed dose per cycle was 5.3 ± 2.1 Gy (0.81 ± 0.32 Gy/GBq). Interestingly, in patients with only a single functioning kidney, ^177^Lu-PSMA-617 RLT was well tolerated, without any signs of acute or subacute nephrotoxicity during a mean follow-up of nearly 2 years.

### 2.7. Adverse Effects and Suboptimal Treatment in Targeted Somatostatin Receptor and PSMA Radionuclide Therapy

Both RLT in PCa and PRRT in GEP-NETs remain noncurative. Thus, the prolongation of life must be balanced against the direct toxicities of the treatment and their impact on quality of life. Today, at least in PRRT and potentially in RLT, many patients are undertreated with the current therapeutic regime considering the relatively low toxicity seen in the kidneys and bone marrow, which are considered the dose limiting organs [57,58].

The uptake of radiolabeled small molecule-based PSMA radiopharmaceuticals is high in the salivary glands [59,60]. Consequently, xerostomia is among the most common side effects, particularly for α-therapy with, e.g., ^225^Ac-PSMA, whereas ^177^Lu labeled PSMA rarely leads to symptoms [61]. However, certain risk factors for nephrotoxicity with ^177^Lu-PSMA-617 including age, hypertension, and pre-existing kidney disease have been reported [62].

There are several ways to mitigate the potentially negative side-effects of PRRT or RLT. Frequently used kidney protection during PRRT and PSMA targeted radionuclide therapy involves the administration of amino acids, mostly composed of the positively charged amino acids l-Lysine and l-arginine, to reduce the uptake and retention of radiolabeled peptides in the kidneys [45,63,64]. The strategy effectively reduces the kidney uptake and therefore the absorbed dose to the kidneys [65]. However, it affects electrolyte balance and can give rise to vomiting and nausea and to hyperkalemia, a life threating state [64]. Besides the well-established regimen of fractionated therapy, which keeps the absorbed dose lower at each infusion and allows normal tissue to recuperate, and the administration of amino acids, additional more experimental strategies to protect kidneys and potentially other normal tissue exist, e.g., Amifostine, blockers of the renin-angiotensin-aldosterone system (RAAS), metformin, and Glutamate Tablet Administration in PSMA RLT [66,67,68,69].

However, if radioprotection of the kidney is to have any beneficial outcomes for the therapy of the patient, in addition to lowering the activity uptake in it, it would be by allowing a higher absorbed dose threshold for the kidney. For this benefit to be effectively realized, it is important that first a new MTD is found and, second, that the treated patients receive individual dosimetry allowing administration of highest possible activity below the MTD. This approach could potentially be applied to both radionuclide therapy in PCa and in GEP-NETs. A 2017 study showed that using individualized dosimetry, more fractions of treatment could be given to patients [26]. Similarly, a study in 2019 showed that increasing the injected activity is feasible from a safety perspective and that the therapeutic outcome was improved using a personalized PRRT approach [58]. The common-place procedure of today, where each patient gets a standard amount of activity and with no individual dosimetry, leads to lower than optimal absorbed doses to the tumor, while not considering the possibilities of radioprotection.

### 2.8. Antioxidants and Radiotherapy

The use of antioxidants in cancer treatment is a controversial topic [70,71,72,73,74]. Due to the oxidative nature of cancer treatments, e.g., radiation and some chemotherapeutics, it is discussed if antioxidants may reduce the therapeutic effect and thereby not only protect healthy cells but also cancer cells. The opposing hypothesis states that antioxidants may improve both therapy and reduce toxicity to normal tissue by reducing the oxidative stress.

Studies with dietary antioxidant supplements such as Vitamin C and E, and carotenoids have been inconclusive, where some have shown benefits for the patients and others even increased adverse effects [75,76]. Importantly, results may differ greatly between antioxidants used, and data most probably cannot be extrapolated; what works with one antioxidant may not work with another. Moreover, dose regimens, combinations of different antioxidants and the differences between dietary and endogenous antioxidants should also be considered carefully, and there is a need of more clinical studies to investigate this in depth.

To overcome issues and improve outcome, the antioxidant may either be selective for non-tumor tissue (e.g., by specific binding or increased bioavailability) or have anti-tumorigenic effect. Another possibility is to have different mechanisms of action depending on site. For example, dephosphorylation (which enables cellular uptake) of Amifostine is influenced by pH levels; it therefore occurs at a higher rate in normal tissue than in the more acidic environment in tumor tissue [77].

The use of Amifostine has shown that ROS and free radicals resulting from ionizing radiation are a potential therapeutic target [68]. Amifostine rapidly accumulates in several tissues, including kidneys, bone marrow, and salivary glands but not to the same extent in tumor tissue [78]. After dephosphorylation, Amifostine can be taken up by cells. Inside the cell, its radioprotective effect is complex and not fully understood, but most likely includes free radical scavenging by its free thiol metabolite [77]. Other possible mechanisms of action include condensation of DNA to reduce area exposed to free radicals and inhibition of DNA repairment in tumor cells [79,80]. For more than 20 years, Amifostine has been FDA-approved for usage against toxicity associated with radiotherapy with reduction in xerostomia being the foremost clinical benefit [81,82].

Similarly to Amifostine, A1M has an active thiol that can scavenge radicals but possesses a more specific occurrence in the kidneys [83] and may therefore be a better therapeutic option for kidney protection (see below).

## 3. α1-Microglobulin

### 3.1. Structure

**Figure 2:**
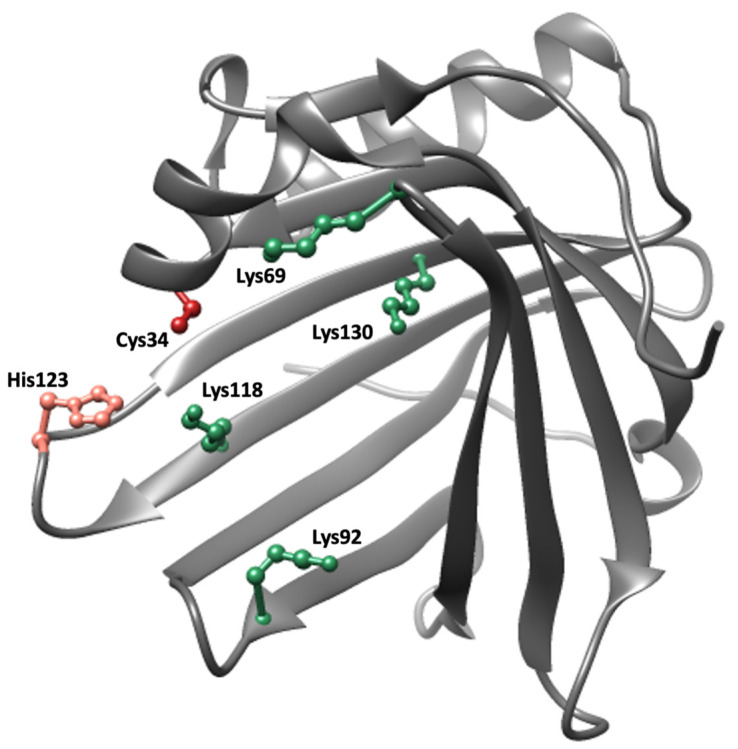
Crystal structure of A1M [87]. Important side chains involved in the protective properties of A1M are highlighted; Cys34 (red), His123 (pink) and Lys69, Lys92, Lys118 and Lys130 (green).

A1M is a small, 26 kDa, glycoprotein consisting of a 183-amino acid polypeptide with two N-linked and one O-linked glycan chains. It belongs to the Lipocalins, a family of approximately 50 structurally related proteins found in vertebrates, plants, and bacteria with a hypothetical common ancestor [84]. The lipocalins are one-domain proteins which consist of a single polypeptide with 160–190 amino acids folded into an 8-stranded beta barrel with a closed bottom and an open end at the top. The interior of the barrel, forming the lipocalin pocket, is usually hydrophobic, and most lipocalins have binding sites for small lipophilic ligands [85]. The archetype lipocalin is retinol-binding protein (RBP), a vitamin A-transporting plasma protein [86]. The three-dimensional crystal structure of human A1M is illustrated in Figure 2 [87]. An unpaired cysteine residue located in a short helix on an omega loop near the opening of the barrel, and two suggested heme-binding sites inside the pocket and between two loops at the opening of the pocket, respectively, are notable features of the structure [88].

### 3.2. Expression

A1M has been identified in more than 60 vertebrate species including fish and birds [9]. In humans, all nucleated cells seem to have the capacity to synthesize A1M, but the hepatocytes of the liver are the major sites of synthesis and secretion of the protein [89,90]. The expression of A1M is regulated by the transcription factor system Keap1/Nrf2 common to most antioxidant proteins [91,92,93], and increased expression of the protein is seen during conditions of oxidative stress and in the presence of ROS, hemoglobin, and free heme [90,94]. An unusual genetic construction codes for A1M in all cells. A precursor protein, the α_1_-microglobulin-bikunin precursor (AMBP), is translated in the ER but cleaved in the Golgi, and the two proteins A1M and bikunin are then secreted separately [95,96]. Bikunin is a protease inhibitor and extracellular matrix component [97] and has no known common function with A1M after secretion. This genetic construction is conserved in all species where A1M and bikunin have been detected, but the biological significance of their co-synthesis is still not fully understood. However, recent results in A1M knockout mice suggest that the genetic linkage of A1M to bikunin is crucial for correct folding of the latter [98].

### 3.3. Distribution

**Figure 3:**
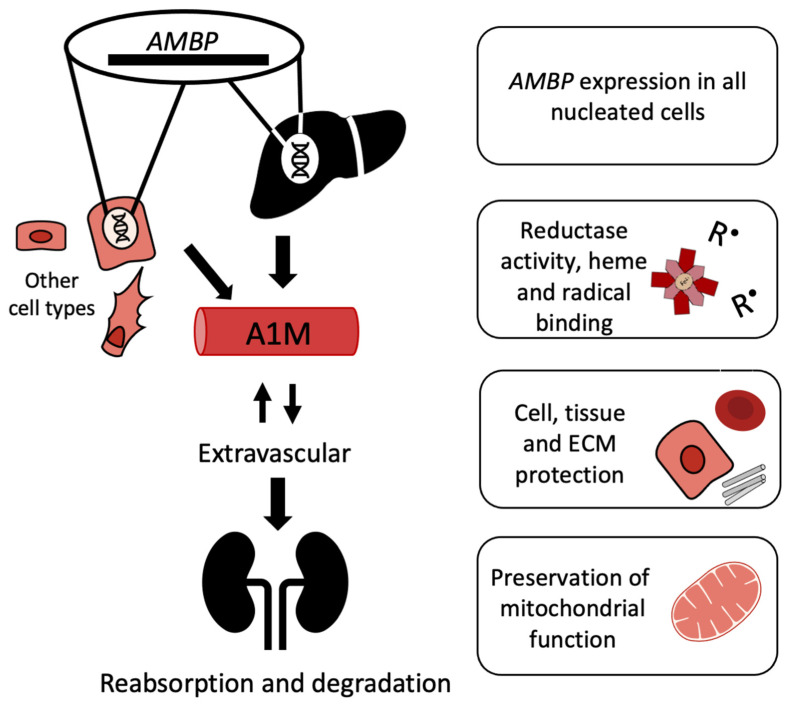
Lifecycle of A1M. Regulation of *AMBP* expression occurs through the Nrf2-pathway and possibly other pathways. Expression occurs in all nucleated cells and increases in response to heme or oxidative stress. The liver is the main site of synthesis, but other cell types also express *AMBP*. A1M is extravasated from the bloodstream and can also be internalized into cells. The antioxidant A1M has reductase activity; can bind heme and radicals; protect cells, tissue, and ECM components; and preserve mitochondrial function. A1M passes through the glomerular membrane and is catabolized in the kidneys.

After secretion to the blood, A1M reaches a concentration of approximately 1–2 µM [99] of which about half is complex-bound to IgA, albumin and prothrombin via disulfide linkages [100]. A1M is rapidly equilibrated between intra- and extravascular compartments, and its half-life in blood is only 2–3 min [101]. Thus, A1M is found in the extracellular fluids of all tissues surrounding epithelial cells. It has been shown to be particularly abundant in extracellular matrix, on cell surfaces, and at blood vessel basal membranes [101], partly due to its affinity for collagen and heparin [102,103]. As a result of its relatively small size, the protein is cleared from the blood circulation by the kidneys through glomerular filtration followed by reabsorption and degradation in the proximal tubular cells (Figure 3) [104].

### 3.4. Molecular Mechanisms

Three different molecular mechanisms have been described for A1M and provide the biochemical basis for its antioxidation protective function: enzymatic reductase activity, radical scavenging, and binding of free heme groups. (1) The reductase activity is dependent on the free thiol group of the Cys34 residue [105]. The reductase activity is catalytic in the presence of either of the electron donating cofactors NADH, NADPH and ascorbic acid, and cytochrome c, methemoglobin, ferric (Fe-III) ions, and carbonyl groups of oxidized proteins are substrates that can be reduced by A1M [105,106]. (2) Radical scavenging activity results in the presence of small free radicals when no electron donating cofactors are present nearby. A molecular mechanism scheme was proposed [107] where (i) the Cys34 thiol group undergoes a one-electron reaction resulting in reduction of the external free radical and oxidation of the Cys34 side group to a thiyl radical, followed by (ii) an intramolecular electron transfer within A1M from a lysyl or tyrosyl side-group yielding reduction/regeneration of Cys34 and generation of a lysyl or tyrosyl radical, and (iii) a reaction between the lysyl/tyrosyl radical and an external free radical resulting in a stable covalent, lysyl or tyrosyl adduct. A1M in urine is always modified with such brown-colored covalent products suggesting that the radical scavenging mechanism is generally active in vivo [108,109]. (3) Two binding sites for free heme-groups have been identified in A1M, one buried in the lipocalin pocket and another more superficially located between two loops at the opening of the pocket [88,110]. Under certain conditions, yet not completely understood, a truncated form of A1M (t-A1M) lacking the C-terminal tetrapepide leucine-isoleucine-proline-arginine is generated from a reaction with hemoglobin or lysed red blood cells. t-A1M is capable of degrading the heme group to a heterogenous chromophore associated with the protein [111]. Free heme groups are strongly toxic and pro-oxidative in a physiological environment due to its chelated iron-atom, and the heme-binding and degradation activities of A1M therefore contribute to its antioxidant protective function.

### 3.5. Mitochondrial Association

A1M was shown to be localized to the mitochondrial membranes in several tissues, cell types, and cell lines [112]. An uptake and import of A1M to the mitochondria and specific localization to Complex I of the respiratory chain was demonstrated. When exposed to ROS and heme-induced oxidative stress, the uptake of A1M and association with mitochondria prevented swelling and loss of function, i.e., ATP-production, of the organelle. The uptake of A1M was increased severalfold immediately before apoptosis, and it was speculated that A1M is involved in protection of mitochondrial energy-production during the initial phases of cell death and apoptosis when local concentrations of ROS and free heme are high [112].

### 3.6. Therapeutic Applications

Based on its molecular mechanisms, biodynamic properties, and protective effects, A1M has been suggested to have therapeutic effects in various diseases associated with oxidative stress and/or hemolysis. Thus, in vivo animal models have been employed to show pre-clinical therapeutic properties in preeclampsia, a pregnancy complication characterized by hemolysis, hypertension and AKI [113], PRRT, i.e., tumor radiotherapy associated with AKI (see below), and intraventricular hemorrhage of prematurely born babies (IVH) which is associated with hemolysis, inflammation and subsequent brain damage [114]. Recently, A1M was shown to have anti-hemolytic properties [115], i.e., A1M could prevent rupture of red blood cells induced mechanically or after exposure to free heme or ROS, and a number of other hemolytic and oxidative stress-related conditions were discussed in terms of possible therapy with A1M-infusion [116].

### 3.7. Recombinant A1M

Production of large amounts of human A1M for therapeutic use can be achieved by recombinant expression in *E. coli* [117]. A mutated variant of recombinant A1M, A1M-035, with higher solubility and stability than recombinant wildtype A1M was recently developed and shown to have identical mechanistic and protective properties [118]. A functional recombinant human A1M was also recently expressed and purified in *N. Benthamiana* tobacco plants [119], a potential alternative source of recombinant A1M for therapeutic use.

## 4. A1M as a Radioprotector

### 4.1. In Vitro

The first indication of the protective role of A1M against radiation induced damage was shown in vitro. Irradiating 0.02% of the cells of a human hepatoma cell line enabled investigation of both directly hit cells but also the surrounding bystander cells (98.98%) [120]. Cell death in directly hit cells and bystander cells both increased and kept on increasing for up to three days with directly hit cells reaching approximately 95% and peripheral cells 50% cell death. Exogenously added A1M reduced cell death completely in the bystander cells and by 50–70% in the directly hit cells.

Several genes encoding antioxidant defense proteins such as heme oxygenase 1, superoxide dismutase, catalase, and glutathione peroxidase 1, as well as cell-cycle regulation, e.g., p21 and p53, showed an increased expression in the same study, and this increase was suppressed in response to added A1M [120]. The hepatoma cells also increased production of A1M in response to radiation, measured both by gene expression and the amount of endogenous A1M in the surrounding media. The induction of several antioxidants, including A1M, further strengthens the idea of oxidative stress as an important aspect of radiation induced damage.

Lipid peroxidation and the presence of protein carbonyl groups, biomarkers of increased oxidative stress [121,122,123], both increased in response to irradiation [120]. However, these parameters were normalized in the presence of exogenously added A1M. The mechanisms described above, reductase and radical scavenging, are most probably involved in the cytoprotective effect of A1M against radiation-induced damage. This was also suggested by using site-directed mutation of the functional Cys34 sidechain (exchanging it for a serin) [106]. The reductase and radical scavenging activities were reduced in the C34S-mutated form of A1M, and in a similar experimental setup as described above, the C34S-A1M mutant showed less protection of irradiated cells compared to WT-A1M.

### 4.2. Biodistribution

**Figure 4:**
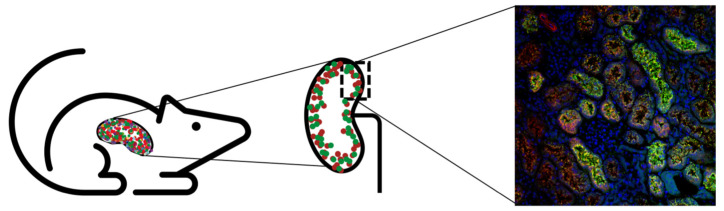
Uptake of A1M and the somatostatin analog octreotate was shown to co-localize on different levels [83]. First, SPECT showed uptake of both molecules in the kidney. Second, autoradiography indicated cortical uptake of both octreotate and A1M. Lastly, colocalization of A1M (green), octreotate (red), and DAPI (blue) was seen in the kidney cortex (magnification 20×).

For A1M to work as a radioprotector in vivo, the protein should be co-localized with the radiopeptides after infusion. A study by Larsson et al. showed that ^125^I-labeled A1M have a short halftime in blood (2–3 min) and predominately localize to the kidneys after i.v. injections in rats after a few minutes [101]. Thereafter, a thorough study was conducted by Ahlstedt et al. where the authors described the pharmacokinetics and biodistribution of intravenously administered A1M and the somatostatin analogue octreotide (Figure 4) [83]. SPECT/CT images displayed prominent uptake in the kidneys for both ^125^I-A1M and ^111^In-octreotide, predominantly in the cortex. The biodistribution study concluded that there was a high renal uptake (46% vs. 75%) of the administered activity for ^111^In-octreotide and ^125^I-A1M, with the peak concentration of A1M after 10 min compared to 20 min after injections for octreotide. The results indicated a rapid renal localization for both molecules but slightly faster for A1M. The kinetics of A1M was further established by measuring injected non-labeled A1M in serum and kidneys, with the highest amounts found after 10 min. Correspondingly, biodistribution studies in mice with ^177^Lu-PSMA-617 showed rapid uptake in the kidneys, with the highest activity at the first timepoint (15 min) [124]. Results suggest that administration of A1M as treatment during PRRT and RLT should be given at the same time or slightly later.

Digital autoradiography further supported foremost cortical localization of ^111^In-octreotide and ^125^I-A1M after 20 and 60 min [83]. Interestingly, images also indicated that the distribution was not homogenous in the cortex. Immunofluorescence microscopy showed strong labeling of both non-labeled A1M and octreotide in cortex, medulla and collecting ducts, with high co-localization in tubular structures as well as intracellularly. The staining was significantly less intense after 4 h post-injections compared to that found 20 min post-injections, suggesting that the clearance occurs quite rapidly of both molecules. A1M was mostly detected in the proximal tubules, which indicates that the recombinant A1M, the possible therapeutic option, is cleared by its natural route [101,125]. Moreover, the two different recombinant versions A1M-WT and A1M-035 showed similar in vivo distribution [118], further supporting that the clearance route is intact.

### 4.3. ^177^Lu-DOTATATE In Vivo Mouse Model

In both patients and mouse models, ^177^Lu-DOTATATE-administration has been associated with kidney damage [11,126]. The nephroprotective effects of A1M were reported in a radiation therapy mouse model, where female BALB/c mice were injected with 150 MBq of ^177^Lu-DOTATATE with or without co-injections of A1M (7 mg/kg) and examined for short- and long-term kidney radiation damage [127]. Animals sacrificed after 1, 4, or 8 days had significantly increased DSB, as detected by immunofluorescence labeling of the DNA damage marker gamma-H2AX in kidney sections. DSB were especially pronounced in the cortex of animals receiving ^177^Lu-DOTATATE, with maximum damage occurring after 4 days. The A1M co-injected animals, however, had significantly less DSB, equivalent to control animals. Most DSB were found in the cortex, consistent with the results in the biodistribution study where the somatostatin-analogues had the highest occurrence [83]. The apoptotic gene response was upregulated already after 1 day in the ^177^Lu-DOTATATE group; however, less so was seen in animals receiving A1M. Taken together, the study concluded that A1M protects against the immediate damage occurring after radiation to the kidneys.

Overall survival was decreased in the long-term ^177^Lu-DOTATATE group, but not in the A1M co-injected animals [127]. Kidney function was measured by albumin in the urine, and this was found to be increased in ^177^Lu-DOTATATE-injected animals, but not in A1M co-injected animals after 6 and 12 weeks. Further supporting the hypothesis that the radiation gives non-reversible kidney damage was the loss of glomeruli and histological kidney damage still seen 24 weeks post-radiation. However, the study reported that animals receiving A1M had less histological damage, with a significantly higher number of functional glomeruli. The expression of stress genes in both liver and kidneys was increased after 6 weeks, suggesting that the stress response may not be limited to the kidneys.

Ideally, as a radioprotector during PRRT, A1M should not interfere with either uptake of radiopeptides in tumor tissue or tumor regression. The biodistribution of ^177^Lu-octreotate was studied in female BALB/c nude mice with established human medullary thyroid carcinoma (GOT2) tumors [128]. The activity concentration in adrenal glands, kidney, lungs, pancreas, blood, femur, liver, spleen, and GOT2 tumors did not differ between groups receiving ^177^Lu-octreotate with or without A1M (5 mg/kg) at any timepoints (1–168 h). The same report also studied the regression and regrowth of human small intestine NETs (GOT1) tumors after treatment with 30 MBq ^177^Lu-octreotate in the presence of A1M. In both groups, radiotherapy induced tumor regression for two weeks followed by tumor regrowth for up to ten weeks. No differences in tumor volumes could be detected during this time, suggesting that A1M does not protect tumor tissue upon administration.

The exact pro-oxidant mechanisms involved in ^177^Lu-DOTATATE-induced renal toxicity are understudied and to our knowledge not many details are known. However, as shown by the mouse study described above [127], cortical DNA-damage was a major effect seen four days after ^177^Lu-DOTATATE administration. DNA-breaks are frequently seen as a result of oxidative damage and exposure to ROS, and A1M should be able to counteract the DNA-damage by employing its radical-binding and reducing mechanisms, which in fact was reported. Another indication of pro-oxidant associated stress in the mouse study was obtained in the upregulation of the kidney Ho-1 and Hsp70 genes after ^177^Lu-DOTATATE administration, both of which were mitigated by co-administration of A1M [127].

### 4.4. ^177^Lu-PSMA-617 In Vivo Mouse Model

Similar to using ^177^Lu-DOTATATE to treat neuroendocrine tumors, ^177^Lu-PSMA-617 is administered to patients to treat metastatic castration resistant prostate cancer. Although significant nephrotoxicity has not been reported so far [51,129], potential kidney damage may be dose-limiting due to the presence of PSMA receptors in healthy kidney tissue and renal uptake. Therefore, a radioprotector such as A1M could be of potential use. In a recent study, ([^99m^Tc]Tc-MAG3) SPECT imaging was used to evaluate kidney function after ^177^Lu-PSMA-617 (100 or 50 MBq) injections in male BALB/cAnNRj mice [130]. From the resulting renogram, perfusion, uptake, and excretion of the radiotracer can be analyzed, as previously reported in a kidney mouse model of unilateral ureteral obstruction [131]. A general decline of these parameters over time was reported, comparing three and six months with baseline. However, the decline seen at three months was less in animals receiving A1M (5 mg/kg with additional 5 mg/kg after 24 h), suggesting that A1M protected the kidneys at medium term; however, the protective effect was not evident after six months [130].

At the same time, measurements of functional urine and serum markers as well as histological examination showed very few differences between the groups receiving ^177^Lu-PSMA-617 and control animals. Dosimetry calculations showed that the mean absorbed dose was 7.36 Gy at 4 days after the 100 MBq injection (3.68 Gy for 50 MBq) to the kidneys. Translating this to the clinical situation, it is roughly in the same range as one fraction given to patients [47,48,49,55], possibly explaining why the traditional kidney damage markers did not detect renal damage.

In the same study, the potential interference of A1M with tumor treatment was investigated in nude mice with subcutaneous LNCaP xenografts [130]. Quantification of ^177^Lu-PSMA-617 in tumors from SPECT/CT imaging showed no significant differences between animals with or without co-injections of A1M. The treatment response of the xenografts was measured as relative tumor volume with the two groups having undistinguishable regression and regrowth phases in response to ^177^Lu-PSMA-617. Similar to data reported with ^177^Lu-octreotate [128], this indicates that A1M, while effective as a kidney radioprotector, does not protect tumor tissue or interfere with the therapeutic effect.

## 5. Concluding Remarks

PRRT and RLT are in general well tolerated in their current form, however, not curative. There are also patient groups at higher risk of renal toxicity at relatively lower absorbed doses. The protection of healthy tissue, especially the kidneys, in radiation therapy is often the limiting factor when administering treatment to patients. Hence, the use of a radioprotector might allow higher activities and better therapeutic outcome in radionuclide therapies. During the recent years, A1M has emerged as a candidate for kidney protection during radiotherapy. The antioxidation properties of A1M can reduce off-target radiation damage in the kidneys without interfering with tumor treatment itself. Clinical studies with A1M to assess kidney protection from reperfusion injuries are ongoing [132], and with successful results of these phase I studies, kidney protection during cancer radiotherapy may be next in line.
